# Virological research history in China: a century of profiling virologists’ contributions and virological innovations

**DOI:** 10.1093/procel/pwae060

**Published:** 2024-11-04

**Authors:** Zhongzhen Wu, Wanying Gao, Kunlan Zuo, QiangYu Xiang, Xiaoya Chen, Lu Zhang, Hao Cheng, Huan Liu

**Affiliations:** University of Science and Technology of China, Hefei 230026, China; University of Science and Technology of China, Hefei 230026, China; University of Science and Technology of China, Hefei 230026, China; University of Science and Technology of China, Hefei 230026, China; University of Science and Technology of China, Hefei 230026, China; University of Science and Technology of China, Hefei 230026, China; Institute of Microbiology, Chinese Academy of Sciences, Beijing 100101, China; University of Science and Technology of China, Hefei 230026, China; State Key Laboratory of Virology, Wuhan 430072, China

Virology, as a branch of microbiology, is a science that studies viruses, covering various aspects of their life activities, such as types, composition, structure, metabolism, growth, reproduction, genetics, evolution, and distribution, as well as their interactions with other organisms and the environment (Flint et al., 2015; Zuo et al., 2024). The scope of virology research is broad, encompassing virus classification, pathogenic mechanisms, genomics, vaccine development, immunology, diagnostics, and virology education and talent training. Significant progress in virology research has greatly promoted advancements in medicine, agriculture, and public health.

The vigorous development of virology in China is inseparable from the group of Chinese virologists (Kang and Rao, 2023). This study traces the history and achievements of virology research in China through the main thread of virologists, presenting the development context and main contributors of Chinese virology and providing valuable historical experience and scientific basis for future virology research (Fig. 1).

**Figure 1.**
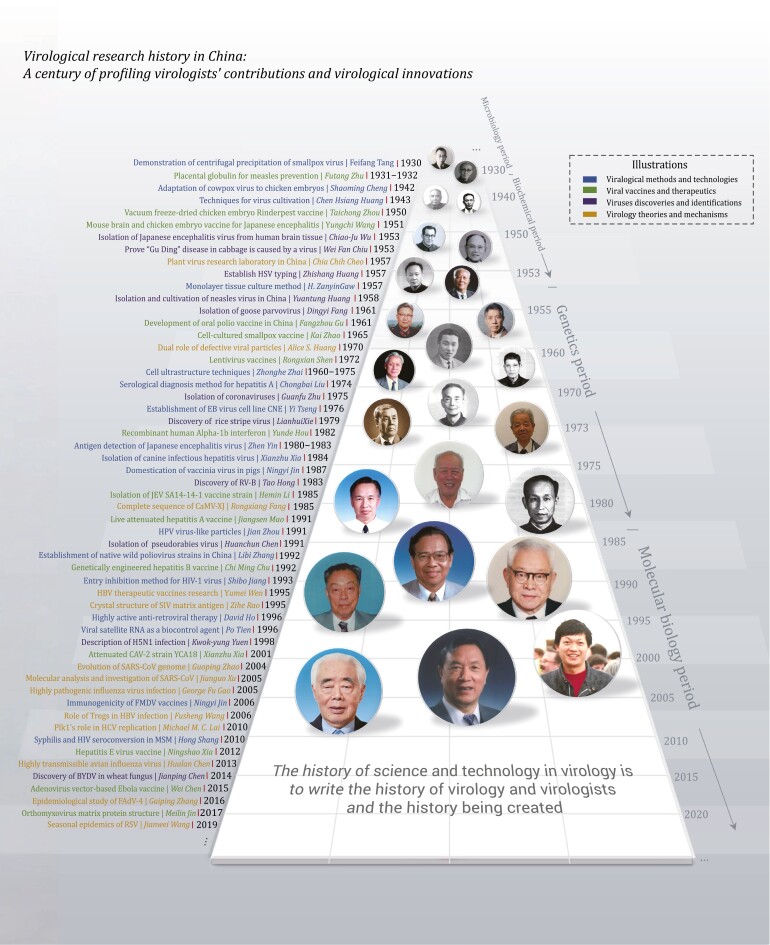
Scientific history of virology and contribution of virologists in China.

## Virological methods and technologies

Fei fang Tang (汤飞凡) was a pioneer in virology in China and globally. During the early days of the electron microscope, he focused on viruses, rickettsiae, etc., and with his mentor Hans Zinsser, he revealed the properties of viruses, such as filterability, centrifugal sedimentation, and self-replication, providing experimental foundations for understanding viral pathogenic mechanisms. Starting in 1922, researchers reported that the vaccinia virus (smallpox) could be separated from pustule fluid using centrifugal sedimentation, but these results were not recognized due to the potential for virus attachment to other particulate matter or cell structures.

This debate continued until 1930, when Tang used a standard centrifuge with a maximum speed of only 4,000 rpm, replacing saline with broth, to centrifuge the vaccinia virus suspension twice at different speeds and verified the filterability of the virus through intradermal inoculation in rabbits, resolving the long-standing controversy (Tang, 1930). Tang was known for isolating the TE8 trachoma “virus” strain, disproving the “bacterial pathogen” theory of trachoma and supporting the “viral pathogen” theory.

Using the yolk sac isolation method, he successfully isolated the world’s first trachoma “virus,” TE8, also known as “Tang’s virus” (Tang et al., 1957), in 1956. To prove its pathogenicity, in 1958, he inoculated himself with the trachoma “virus,” contracted trachoma, collected 40 days of clinical data, and ended the debate over the trachoma pathogen (Tang et al., 1956). Tang was honored as China’s first generation of medical virologists and respected as the “father of chlamydia.” In 1973, the trachoma “virus” was classified as Chlamydia. Additionally, he made significant contributions to vaccine research, developing various vaccines and promoting national smallpox vaccination, leading to the eradication of smallpox in China 16 years ahead of schedule, earning him the title of “father of Chinese vaccines.”

Shaoming Cheng (程绍明) adapted the vaccinia virus to chicken embryos at the North China Institute of Hygiene in 1942, with clinical trials in Shandong and Hebei provinces demonstrating good safety and efficacy. In 1946, he conducted safety and efficacy trials of the 670th generation rabbit-adapted rinderpest virus in Beijing, proving it safe for local dairy cows and effective in the field, which led to its widespread use. He published a monograph on the value of rabbit-adapted rinderpest weak virus for dairy cows, providing a scientific basis for rinderpest prevention (2007).

Chen Hsiang Huang’s (黄祯祥) paper on titration and neutralization of Western equine encephalomyelitis virus in tissue culture marked a milestone in virology research. He pioneered a new virus culture technique involving digesting animal tissues into single-layer cells and inoculating viruses in these cells. Observing pathological changes under a microscope allowed indirect observation and judgment of virus proliferation, laying the foundation for modern virology research, termed the “second technological revolution in medical virology” (Huang and Sanders, 1942; Cheng, 2011). In 1949, Huang began developing Japanese encephalitis vaccines in China. Over the decades, research evolved from initial killed vaccine studies to using tissue culture techniques for attenuated live vaccine development. The achievement won the 1978 National Science Congress Award (Huang et al., 1958).

H. Zanyin Gaw (高尚荫) pioneered research in invertebrate tissue culture and insect viruses by successfully developing a tissue culture method for the silkworm disease virus, a breakthrough achieved in 1957. His work revealed the pathogenic mechanism of the silkworm disease virus and developed control measures, significantly advancing sericulture. He also created the insect cell monolayer culture method, a world-first and a major breakthrough in insect virus research. Under his guidance, Wuhan University’s insect virus research team developed China’s first virus-based insecticide, honored by national departments and winning the Hubei Province Science and Technology Achievement First-Class Award (Liu et al., 1958, 2015).

From the early 1960s to the mid-1970s, Zhonghe Zhai (翟中和) established cell ultrastructure technology in China and developed the first duck plague virus vaccine, making significant advancements in understanding the relationship between animal virus replication and cell structure (Ding and Zhai, 1982; Li et al., 1981). Meanwhile, starting in 1974, Chongbai Liu (刘崇柏) and his team were the first to identify diagnostic hepatitis A antigens in the feces of Chinese acute hepatitis A patients, leading to the development of serological diagnostic methods. This groundbreaking research earned the Chinese Academy of Medical Sciences Scientific Achievement Award in 1979 (Cui and Wei, 1987).

Yi Tseng (曾毅) and colleagues proved in 1978 that highly differentiated cell lines also carry the Epstein-Barr virus (EBV), following their 1976 establishment of the world’s first highly differentiated nasopharyngeal carcinoma cell line (CNE-1). In 1980, he and his colleagues established the first poorly differentiated carcinoma cell line (CNE-2), and in 1987, they established a metastatic nasopharyngeal carcinoma cell line (CNE-3). They subsequently developed early diagnostic methods for nasopharyngeal carcinoma. After decades of research, he ultimately led his team to be the first in the world to prove that EBV is a causative factor in the development of nasopharyngeal carcinoma.

Zhen Yin (殷震) led a team studying the Japanese encephalitis virus from 1980 to 1983, during which they developed a solid-phase reverse complement fixation test system for rapid and highly specific detection of virus antigens (Yin et al., 1980). They also applied enzyme-labeled antibody methods to prove its specificity and sensitivity for primary use. Through 125I-labeled antibody technology, they enhanced detection sensitivity and simplified procedures. He also succeeded in purifying the Japanese encephalitis virus with fish sperm protein, laying the foundation for vaccine and diagnostic reagent preparation and molecular virology research. In 1984, Yin pioneered small RNA virus gene recombination research and established the Military Gene Engineering Laboratory in 1993. He also placed great emphasis on talent cultivation. The virology research lab he established became a renowned brand, producing three academicians of the Chinese Academy of Engineering, including himself, Xianzhu Xia (夏咸柱), and Ningyi Jin (金宁一).

Jian Zhou (周健) and Ian Frazer, in 1991, utilized recombinant DNA technology to create HPV virus-like particles, which eventually led to the first HPV vaccine (Zhou et al., 1991). Released in 2006 by Merck and GlaxoSmithKline, the vaccine marked a public health breakthrough, drastically reducing cervical cancer incidence and mortality and saving millions of lives. Meanwhile, beginning in 1992, Libi Zhang’s (张礼璧) team conducted retrospective studies on the genetic characteristics of indigenous poliovirus strains in China, which resulted in the establishment of a strain bank, a gene database, and a national high-level polio laboratory network (Zhang, 1996). In 1993, Shibo Jiang (姜世勃) and colleagues explored HIV-1 inhibition, discovering that the HIV-1 gp41 protein region 637-666 exhibited strong antiviral activity against various HIV-1 strains without significant cytotoxicity, opening new possibilities for AIDS treatment (Jiang et al., 1993).

Po Tien (田波), in 1996, became the first internationally to successfully apply satellite RNA of viruses as a biocontrol agent against plant viral diseases. He further utilized ribozymes to efficiently inhibit plant pathogens, which led to the development of high-resistance potato lines against viroids. This groundbreaking work offered a new approach to controlling viroid diseases (Tien, 1996). In 1997, he first demonstrated the feasibility of using ribozymes to inhibit viroid replication within plants, providing a new strategy for developing virus-resistant transgenic crops (Yang et al., 1997). In 2001, he demonstrated for the first time that HBV-specific peptides bind to heat shock protein gp96 in hepatocellular carcinoma patients induced by hepatitis B virus (HBV) (Meng et al., 2001). In 2003, he studied the roles of the two heptad repeat regions (HR1 and HR2) in the fusion protein of human respiratory syncytial virus (hRSV) in inhibiting viral fusion. The research results showed that the HR1 and HR2 regions of the hRSV fusion protein effectively inhibit the fusion of the virus with cells. The stable six-helix bundle structure formed by HR1 and HR2 plays a crucial role in blocking viral fusion (Wang et al., 2003).

David Ho (何大一) revolutionized AIDS research in 1996 by developing highly active anti-retroviral therapy, a treatment that transformed AIDS from a fatal disease into a manageable chronic condition. This major breakthrough marked a turning point in the fight against AIDS (Hammer et al., 1997).

Ningyi Jin (金宁一) and his team found in 2006 that the recombinant fowlpox virus vUTAL3CP1, when used alone, induced stronger humoral and cellular immune responses in pigs compared to traditional inactivated vaccines. Their study also demonstrated that combining vUTAL3CP1 with the DNA vaccine pVIRIL18P1 resulted in higher CTL activity. These results highlighted the good immunogenicity of both genetic engineering vaccines and helped identify optimal immunization strategies, providing a foundation for future challenge-protection tests (Jin et al., 2006).

Hong Shang’s (尚红) team studied HIV and syphilis seroconversion in MSM in Shenyang, China, with results from 2010 indicating high prevalence of both diseases in this population. The findings underscored the urgent need for targeted interventions to prevent further transmission (Xu et al., 2010).

## Viral vaccines and therapeutics

Pediatrician Futang Zhu (诸福棠) discovered placental gamma globulin for preventing measles while studying at Harvard Medical School, hypothesizing that antibodies produced in mothers who had recovered from measles were passed to their children through the placenta, granting immunity. After conducting multiple trials, he successfully developed placental gamma globulin, which was used to inoculate children exposed to measles, providing passive immunity or reducing symptoms and lowering the risk of measles-related pneumonia (2018).

Taichong Zhou (周泰冲) and his colleagues in 1950 successfully developed the vacuum freeze-dried chicken embryo rinderpest vaccine. This vaccine demonstrated excellent efficacy in protecting livestock from rinderpest virus infection. Experimental results showed that animals vaccinated with this vaccine developed strong immunity against the rinderpest virus and that the vaccine was highly safe. The freeze-drying technique significantly extended the vaccine’s shelf life, allowing it to remain effective at room temperature for extended periods. This feature made the vaccine more convenient and economical for transportation and storage. The application of this vaccine in several regions of China proved its effectiveness in controlling rinderpest outbreaks (Zhou and Yu, 1950). In 1954, Zhou and collaborators also successfully developed a rabbit-adapted attenuated vaccine strain for classical swine fever, which was awarded the National Invention Prize, the National Science Conference Award, and the Ministry of Agriculture’s Patriotic and High-Yield Award (Zhou, 1958).

Yungchi Wang (王用楫) began developing a mouse brain vaccine for Japanese encephalitis, later advancing to develop a chicken embryo vaccine in 1951. He participated in Japanese encephalitis prevention efforts in Beijing, vaccinating 200,000 children and significantly reducing the disease’s incidence. These achievements were made between 1950 and 1952 (Wang et al., 1953).

Starting in 1953, multiple cases of poliomyelitis were reported in Nantong, Shanghai, Jinan, Qingdao, and other cities in China, with an incidence rate as high as 36–53 cases per 100,000 people, posing a severe situation. Fangzhou Gu (顾方舟) and his colleagues, entrusted by the Ministry of Health, went to the Soviet Union to study the production process of the inactivated polio vaccine. After careful consideration, Gu boldly proposed to the Chinese Academy of Medical Sciences to adopt the live vaccine route. Subsequently, based on their own production, testing, and trial use of the live vaccine among the population, they formulated the “Polio Live Vaccine Manufacturing and Testing Regulations,” which were submitted to and approved by the Ministry of Health in 1964. In China, to eradicate poliomyelitis, it was necessary to ensure that children in vast rural areas could take the live vaccine. However, at that time, the liquid vaccine required dilution before administration, and the diluted vaccine quickly lost its efficacy at room temperature. To solve this problem, in 1961, he led his team to develop the oral polio vaccine, also known as the “sugar pill vaccine.” In 1981, he began research using monoclonal antibody hybridoma technology for the polio virus. In 1982, his team successfully developed the “Polio Monoclonal Antibody Kit.” On July 21, 2000, the National Polio Eradication Certification Committee held the “China Polio Eradication Certification Report Signing Ceremony” at the Ministry of Health, where Gu, along with other members, signed the report. Since then, China has been recognized by the World Health Organization as a polio-free country. His work not only saved countless children from paralysis but also made China one of the first countries in the world to eradicate poliomyelitis, significantly contributing to the global eradication of polio. For this reason, It is affectionately known as the “Grandpa Sugar Pill” (Wang, 1965; Wu et al., 2020).

Kai Zhao (赵铠) and his team successfully adapted the vaccinia virus to chicken embryo cells through two to three passages for vaccine preparation, an achievement made in 1965. Animal experiments demonstrated good immunogenicity and stability. After extensive human observation, they successfully developed a new type of cell-cultured smallpox vaccine. This vaccine was approved in 1969 and subsequently promoted for use by biological product institutes nationwide. It improved vaccine quality, reduced vaccination reactions, and significantly lowered production costs, making a substantial contribution to the prevention and eradication of smallpox (Jian, 2006).

Equine infectious anemia (EIA), caused by the EIA virus, has a high mortality rate and has caused significant losses in the horse industry. Rongxian Shen (沈荣显) proposed a research method using donkey leukocytes to cultivate and attenuate the virulent strain of the virus, pioneering the Chinese approach to EIA research. Starting in 1972, he serially passaged the virulent EIA strain in donkey leukocytes for 125 generations over more than a decade, changing the virus’s growth conditions and obtaining a live attenuated vaccine with good immunogenicity, breaking through the barrier of lentivirus immune prevention. Since 1975, this vaccine has been promoted for practical use, with over 1 million equids in China vaccinated. With the vaccine’s application, the incidence rate of the disease has significantly decreased, and currently, almost no cases occur. EIA has been effectively controlled and prevented in China. To date, this vaccine remains the only lentivirus vaccine widely used on a large scale worldwide (Yuan et al., 1957).

Yunde Hou (侯云德) and his team successfully cloned the human alpha-1b interferon gene, possessing independent intellectual property rights, and developed China’s first gene-engineered drug, recombinant human alpha-1b interferon. This achievement marked a breakthrough in China’s gene engineering drug development, accomplished in 1982. This pioneering work ushered in the era of gene engineering drugs in China. As early as 1977, the United States had successfully applied genetic engineering technology to produce growth hormone-releasing factor. Upon learning of this news, Hou was greatly inspired and keenly recognized the potential of this promising new technology. Subsequently, he led his team in meticulously studying all accessible literature and gradually progressing through trial and error. By 1979, they had extracted interferon mRNA from over ten thousand milliliters of human blood leukocytes induced by the virus. After extensive searching, they replaced the originally intended African clawed frog oocytes with African carp oocytes from a Beijing farm, further determining this mRNA and establishing the interferon mRNA translation system in African carp oocytes. Overcoming equipment shortages and other challenges, the team finally achieved this significant result in 1982 (Wu, 2018).

Hemin Li (李河民), Yongxin Yu (俞永新), and their team significantly improved the immunogenicity of the Japanese encephalitis vaccine by using a method of continuous subcutaneous passage in suckling mice, an accomplishment achieved in 1985. Through plaque reduction neutralization tests, they screened out the highly attenuated, stable, and highly immunogenic strain SA14-14-1. This strain was used for the production of the live attenuated Japanese encephalitis vaccine, which was first produced in 1987, a pioneering achievement both domestically and internationally. The widespread application of these vaccines demonstrated a protective efficacy of 80%–90%, making it one of the top ten scientific and technological achievements in China in 1989, and in 1990, it was awarded the National Science and Technology Progress Award (Li and Yu, 1959).

In the late 1970s, Jiangsen Mao (毛江森) isolated the hepatitis A virus (HAV) and discovered that rhesus monkeys and cynomolgus monkeys could be infected with HAV and had immune responses, establishing an animal model and proving the existence of subclinical hepatitis A infections. Subsequently, he led a team to successfully isolate China’s first strain of HAV (H2M20K5, referred to as the H2 strain). In 1991, based on the H2 strain, they developed the world’s only live attenuated hepatitis A vaccine (H2 strain). Clinical use of this vaccine proved it to be safe and effective, leading the world in this field. In 1993, it was awarded the National Invention Second Prize, the Ministry of Health Science and Technology Progress First Prize, and the Zhejiang Province Science and Technology Progress First Prize. It also received a patent certificate from the National Patent Office. In 1995, this invention was recognized as one of the top 10 news stories in China’s science and technology community. Since then, the vaccine has been used by over 120 million people and has been exported to countries such as India, making a significant contribution to the global control of hepatitis A (Liu et al., 2024).

Chi Ming Chu (朱既明), in 1950, became the first in China to observe the morphological diversity of the influenza virus. He also played a key role in the successful development of various vaccines, including the live attenuated measles vaccine, the mammalian cell-based genetically engineered hepatitis B vaccine, and multivalent vaccines using the vaccinia virus as a vector. In 1992, after more than a decade of effort, he and his colleagues successfully launched the genetically engineered hepatitis B vaccine expressed in mammalian cells, the world’s first of its kind. This vaccine provided an important tool for the prevention and control of hepatitis B in China. He also led the team that developed the world’s first vector system using vaccinia virus gene expression. Using this system, they completed Phase I clinical studies of five recombinant vaccinia virus vaccines for diseases such as hepatitis A, hepatitis B, measles, and EBV infection (Qing, 2017).

Ningshao Xia (夏宁邵) has led a team in researching a proprietary *E*scherichia* coli* prokaryotic expression virus-like particle vaccine technology system, an effort that began in 2001. In 2012, the first research outcome based on this technology—the world’s first hepatitis E virus (HEV) vaccine, Hecolin—was approved for market release. This vaccine was the first in the world to be approved for the prevention of HEV infection (Cao et al., 2018; Zhu et al., 2010). In 2019, another research outcome based on this technology—the first domestically produced recombinant human papillomavirus (HPV) vaccine, Cecolin—was approved for market release. This vaccine made China the third country in the world, after the United States and the United Kingdom, to achieve an independent supply of cervical cancer vaccines.

Xianzhu Xia (夏咸柱) and his team conducted experimental immunization studies on the naturally attenuated type II canine adenovirus strain YCA18, with this work being completed in 2001. The results showed that the YCA18 strain exhibited good safety and immunogenicity in dogs, effectively protecting them from virulent CAV-1 infection. Furthermore, the YCA18 strain demonstrated genetic stability in passage experiments across different animals and cell lines and showed no immunological interference when used in combination with other vaccines (Xia et al., 2001).

Wei Chen (陈薇) and her team demonstrated in 2015 that the novel recombinant adenovirus type 5 vector-based Ebola vaccine showed good safety and immunogenicity in healthy adults. Their research indicated strong antibody and T-cell responses in both low- and high-dose groups, with primarily mild to moderate adverse reactions. The study highlighted the vaccine’s potential for responding to Ebola outbreaks and called for further large-scale clinical trials to assess its long-term safety and protective efficacy (Feng-Cai et al., 2015). Two years later, in 2017, Meilin Jin (金梅林) and his team elucidated the crystal structure of the matrix protein from the orthomyxovirus infectious salmon anemia virus (ISAV). They revealed mechanisms for self-polymerization and membrane association, showing that the ISAV matrix protein forms a two-dimensional lattice through interactions between its N and C termini and binds to membranes via electrostatic interactions mediated by a positively charged surface loop. These findings provided critical structural insights into the role of orthomyxovirus matrix proteins in virus assembly and budding processes (Zhang et al., 2017).

## Viruses discoveries and identifications

Chiao-Ju Wu (吴皎如) isolated the Japanese encephalitis virus from encephalitis patients’ brain tissue, providing a foundation for further research, and proposed effective control measures, including vector control and diagnostic methods, an achievement made in 1953 (Wu and Wu, 1956). In the same year, Wei Fan Chiu (裘维蕃) resolved a long-standing debate by proving that the “solitary plant disease” in cabbage was caused by a virus, with aphids as the vector. In the late 1950s, he investigated the beet yellows virus in Inner Mongolia, confirming its transmission by aphids and suggesting control measures. His contributions continued into the 1980s, when his research on wheat dwarf disease earned him the Science and Technology Progress Award from the Ministry of Agriculture and the State Science and Technology Commission. In 1983, his team developed an anti-viral agent (“83 increase resistance agent”) for crops, which won a First-Class Award from the National Education Commission and a State Natural Science Third-Class Award, leading to widespread agricultural application. Additionally, he introduced a plant virology course at Beijing Agricultural University, authored China’s first plant virology textbook, and established a plant virology laboratory (Qing, 2011a).

Human herpesvirus (HSV) now has two types: HSV-1 and HSV-2. Before 1957, there was no concept of HSV typing. The classification of HSV owes much to the research of Chinese virologist Zhishang Huang (黄志尚), one of the world’s first scientists to identify the two types of herpes simplex virus. In 1957, he isolated multiple virus strains from recurrent herpes lesions and discovered differences in their antigenic and biological characteristics, confirming and naming them HSV-1 and HSV-2. These virus strains became the international reference strains for herpes simplex virus types 1 and 2. This discovery laid the foundation for the classification of the herpes simplex virus and provided important scientific evidence for subsequent herpesvirus research (Shubladze and Khuan, 1959).

In 1958, Yuantung Huang (黄元桐) was the first in China to isolate and culture the measles virus, laying the groundwork for the development of the measles vaccine (Qing, 2011b).

In 1956, during an investigation of gosling deaths in Yangzhou, Dingyi Fang (方定一) discovered and preliminary identified the disease as a new viral infectious disease, naming it “gosling plague.” In 1961, he successfully isolated the virus and confirmed that it could be passaged in duck and goose embryos and retained its pathogenicity after being stored in a refrigerator. In 1962, he prepared antiserum by inoculating mother geese with the virus, achieving a protection rate of 86.7%–92%. In 1963, he successfully controlled the gosling plague epidemic in Jiangsu Province using high-titer antiserum and developed a vaccine, which was subsequently promoted nationwide (Qing, 2013).

Guanfu Zhu (朱关福) led a team that investigated the infection rate of coronaviruses in the population, an effort that began in 1976. By 1979, his team had isolated strains of coronaviruses, rhinoviruses, and adenoviruses, establishing a strong foundation for the etiological study of respiratory infectious diseases in China (Zhu, 1958, 1979). In 1987, Zhu’s team also isolated China’s first human immunodeficiency virus (HIV) strain from the blood of AIDS patients (Sun et al., 1991). Around the same time, in 1979, Lianhui Xie (谢联辉) discovered and began researching rice dwarf disease. After years of fieldwork and laboratory analysis, he confirmed that the disease was caused by a new virus, which he named the rice dwarf virus. His research clarified the physicochemical properties of the virus and established it as a new member of the plant reoviruses. Xie also proposed effective control measures and discovered two new rice viruses in China—rice gall dwarf virus and rice black-streaked dwarf virus—as well as a new vector for rice dwarf disease, the small brown planthopper (Li, 1985).

Tao Hung (洪涛) made a groundbreaking discovery, identifying the human group B rotavirus (RV-B), the first in the world to do so, with systematic molecular biological research following in 1983 (Hung et al., 1983a). In the same year, he also discovered the morphology of the nephropathia epidemica hemorrhagic fever virus, resolving long-standing pathogenetic questions and significantly advancing research in this field (Hung et al., 1983b). Nearly a decade later, in 1991, Huanchun Chen (陈焕春) and his colleagues isolated the pseudorabies virus from an outbreak of stillbirths in sows, marking the first identification of a pseudorabies outbreak in pigs in China. Following this, Chen’s research identified the five major clinical symptoms of pseudorabies in pigs, developed vaccines, and proposed an eradication plan for pseudorabies in the country (Chen et al., 1991).

Kwok-yung Yuen (袁国勇) and his colleagues in 1998 provided a detailed account of the clinical manifestations of 12 human H5N1 infection cases and identified risk factors leading to severe illness. Their research demonstrated that H5-specific RT-PCR was the most effective method for rapidly detecting the H5N1 virus, emphasizing the high complication rate of the infection and the importance of early diagnosis and treatment to improve prognosis (Yuen et al., 1998). In 2014, Jianping Chen (陈剑平) made a significant discovery by identifying barley yellow mosaic virus and barley yellow dwarf virus within *Polymyxa graminis*, providing the first direct evidence of fungal transmission of plant viruses. His work revealed the intricate relationship between fungi and the viruses they transmit, while also elucidating the occurrence patterns and pathogenic mechanisms of five key rice and wheat viruses. The green control technologies Chen developed for these diseases have since been widely applied in affected regions of China (Chen et al., 2014).

## Virology theories and mechanisms

Chia Chih Cheo (周家炽), in 1957, established a plant virus research group at the Institute of Applied Mycology of the Chinese Academy of Sciences (now the Institute of Microbiology), building on the earlier work of Chuanguang Lin, Dafu Yu, and Wei Fan Chiu. A year later, the Institute of Microbiology was established, forming China’s first plant virus research laboratory. During his time at the California Institute of Technology, he studied the proliferation mechanism of the tobacco mosaic virus in tobacco leaves and discovered the correlation between the growth of the tobacco mosaic virus and the decrease in the major protein levels of the cytoplasm. This result was published in the Journal of Biological Chemistry in 1949 and was one of the early international studies on the biochemistry of viruses and their relationship with hosts (Qing, 2010).

Alice S. Huang (黄诗厚) and her colleagues in 1970 were the first to systematically describe the dual role of defective viral particles in viral infections. They found that these particles could protect the host by interfering with the replication of normal viruses, while under certain conditions, they could also enhance viral pathogenicity. These findings significantly contributed to understanding the complexity of viral infections and viral pathology, providing a scientific basis for future antiviral strategies (Huang and Baltimore, 1970). In 1985, Rongxiang Fang (方荣祥) published the complete genome sequence of China’s first plant virus, the Xinjiang strain of cauliflower mosaic virus (CaMV), and further advanced plant virus genomics by cloning and sequencing the coat protein genes of various plant viruses. His work also led to the development of transgenic tobacco plants resistant to tobacco mosaic virus and cucumber mosaic virus. These transgenic plants were widely cultivated by 1992, representing one of the largest cases of transgenic plant cultivation in the world at the time (Fang et al., 1985).

Yumei Wen (闻玉梅), as one of the pioneers of therapeutic hepatitis B vaccines in China, has been dedicated to HBV research for many years. She was the first to propose the new therapeutic concept of “eliminating immune tolerance to HBV antigens.” Based on this, she established an animal model that simulates immune tolerance to hepatitis B in young children and designed five therapeutic vaccines to eliminate immune tolerance. In 1995, she and her team published a paper that first proposed the concept of therapeutic vaccines and introduced the progress of this work in China (Wen et al., 1995). This groundbreaking paper has since been frequently cited by researchers in the field.

Zihe Rao (饶子和) and his team, in 1995, elucidated the crystal structure of the simian immunodeficiency virus (SIV) matrix antigen, uncovering its crucial role in virus assembly. Their high-resolution analysis revealed unique folding patterns and surface features of the matrix antigen that are essential for the formation and stability of the viral capsid, offering important molecular insights into the assembly mechanisms of SIV and related viruses. These findings also provided potential targets for antiviral drug design (Rao et al., 1995). In 2004, Guoping Zhao (赵国屏) and his colleagues investigated the genomic variation and evolution of the SARS coronavirus during its outbreak in China. Their research showed that the virus underwent both positive and purifying selection as it adapted to human hosts, supporting the hypothesis that SARS likely originated from wild animals and evolved during its adaptation to humans (Chinese SARS Molecular Epidemiology Consortium, 2004).

Jianguo Xu (徐建国) and his team in 2005 made significant contributions to understanding the molecular evolution and host identification of the SARS virus. Their research uncovered the mechanisms behind cross-species transmission and identified key mutation sites. Xu’s team also developed real-time RT-PCR diagnostic technology, significantly enhancing the speed and accuracy of SARS virus detection and providing vital scientific evidence and strategic support for the prevention and control of the SARS epidemic (Kan et al., 2005). That same year, George Fu Gao (高福) led a team that became the first to confirm that wild migratory birds could be collectively infected with the highly pathogenic H5N1 avian influenza virus. Their study overturned the previous belief that migratory birds were merely reservoirs of the virus. The team reported an outbreak of the H5N1 avian influenza virus in the Qinghai Lake region of China between May and June 2005, demonstrating that the virus posed a potential global threat. Their findings indicated that the virus likely originated from birds wintering in Southeast Asia and spread to Qinghai Lake via migratory bird movements (Liu et al., 2005).

Fusheng Wang (王福生) led a team to explore the role of CD4^+^CD25^+^ regulatory T cells (Tregs) in the circulation and liver during HBV infection, focusing on their impact on the antiviral immune response and disease progression, with this research conducted in 2006. The research results indicated that CD4^+^CD25^+^ Tregs play a crucial role at different stages of HBV infection. They not only regulate immune responses in peripheral blood but also function within the liver, influencing the progression of hepatitis B. The increase in Treg frequency is closely associated with viral replication and liver inflammation in chronic HBV infection and may serve as a potential prognostic factor for disease progression (Xu et al., 2006).

Michael M. C. Lai (赖明诏), in 2010, was the first to systematically reveal the critical role of Polo-like kinase 1 (Plk1) in the replication of hepatitis C virus (HCV), particularly by regulating the hyperphosphorylation of non-structural protein 5A (NS5A). This discovery provided new insights into HCV treatment and laid the groundwork for a deeper understanding of virus-host cell interactions (Chen et al., 2010). In 2013, Hualan Chen (陈化兰) and her team conducted a comprehensive investigation into the cross-species transmission of H7N9 and H5N1 viruses. Their research revealed that H7N9 could be transmitted among ferrets via respiratory droplets, underscoring the significant risk posed by live poultry markets as transmission sources. Meanwhile, they found that H5N1, after reassorting with genes from the 2009 H1N1 virus, showed efficient transmission among Guinea pigs, with the PA and NS genes playing key roles. These findings highlighted the substantial cross-species transmission potential of these viruses, emphasizing the need for enhanced surveillance and control measures to prevent future influenza pandemics (Zhang et al., 2013a, 2013b).

Gaiping Zhang (张改平) and his team in 2016 studied the molecular epidemiology of type 4 avian adenovirus (FAdV-4) strains linked to hydropericardium syndrome (HPS), a viral disease that had been causing outbreaks in central China since 2015. Characterized by hydropericardium and hepatic necrosis, HPS severely affects broiler chickens, leading to significant economic losses. Their research revealed a close genetic relationship between the Chinese strains and earlier strains from India, with mutations likely triggering the sudden outbreak of HPS. This study provided a critical molecular basis for the development of vaccines to prevent and control HPS in the future (Zhang et al., 2016). In 2019, Jianwei Wang (王健伟) led a team to investigate the seasonal epidemic characteristics of respiratory syncytial virus (RSV) in Beijing from 2007 to 2015. Their findings highlighted distinct seasonal patterns in RSV infections, with significant differences between the epidemic seasons of RSV groups A and B. This research offered valuable insights for predicting the start and duration of RSV epidemic seasons, helping optimize prevention strategies and vaccination timing (Yu et al., 2019).

## Conclusion

This study reviews the nearly century-long development of virology in China, highlighting the outstanding contributions and scientific achievements of virologists in various fields, including virus technology and methods, virus vaccines and drugs, virus discovery and identification, and virus theory and mechanisms. The development of virology in China is not only a reflection of scientific and technological progress but also a testament to the wisdom and dedication of countless virologists.

In the development of virology in China, early technologies and methods were in the biochemical period of virology history. Chinese scientists used virological methods to promote the study of the properties of viruses that made viral vaccines and anti-viral drugs, as well as the discoveries and identifications of viruses, thereby deepening pathogen research. With the advancement of science and technology, virology has continually made breakthroughs in research theories and mechanisms by Chinese scientists. This progress not only reflects virology development in China catching up to worldwide advances but also demonstrates that it has gradually reached the forefront of sciences and technologies in its ongoing development.

By reviewing the outstanding contributions of Chinese virologists, we can trace the paths of scientific discovery and technological innovation and understand how scientific breakthroughs have driven the development of virology. Virology history of science and technology studies provides valuable insights for current and future science innovations and also provide knowledge for science education, inspiring public interest in science. This paper reviews the contributions of Chinese virologists in responding to major infectious diseases, developing vaccines, and antiviral drugs; showcases the historical significance and scientific values of virology in China; and provides an important science reference for virology and life sciences.

The development of virology in China has advanced alongside the history of microbiology and biochemistry in the scientific history of virology. Chinese virologists, along with virologists worldwide, have laid the foundation for the establishment of virology. Chinese virologists have always upheld the scientific spirit of being dedicated to people’s health and well-being, serving national needs, integrating theory with practice, and embodying the qualities of perseverance, courage in facing challenges, confidence, and self-reliance. Chinese virologists are keen learners, adept at absorbing experiences and transforming them into innovations. As a result, Chinese virology holds an important position among the leading fields of global virology.

Nowadays virological research in China is marked by ongoing advancements, with scientists contributing significantly to the virology developments, including Zihe Rao, George Fu Gao, Hualan Chen, Zhigang Tian, Zhengli Shi, Zhenghong Yuan, Ke Lan, Guizhen Wu, Chengfeng Qin, Gong Cheng, Xi Zhou, Lu Lu, etc. Their contributions advance the progression of Chinese virology and play a vital role in the advancement of the global virological landscape.

As we reflect on the illustrious legacy of virology in China, virologists emerge as guiding lights in the extensive history of this field. Moving forward, the contributions of China’s virologists, both highlighted and unmentioned in this paper, collectively shape the past, present, and future of virology.
